# Supplementary material for: Unveiling the complexity of spatiotemporal soliton molecules in real time
Source: Nat Commun. 2023 Apr 11;14:2029. doi: 10.1038/s41467-023-37711-6 (PMC10090195; doi:10.1038/s41467-023-37711-6)
Supplement: Supplementary file 1 — Supplementary Information [file 41467_2023_37711_MOESM1_ESM.pdf]

Supplementary Information for:

Unveiling the complexity of spatiotemporal soliton molecules in real time

Guo and Lin et al.

Supplementary Note 1: Numerical simulation of 3D soliton molecules

In our numerical studies, we consider a simulation model that consists of a grade-index (GRIN) gain fiber (50 cm length, 62.5 μm core diameter), an artificial saturable absorber, a beam splitter, a bandpass filter, and a space filter. Note that, to speed up the simulation, here all the parameters of the fiber laser cavity, including gain, nonlinearity, chromatic/modal dispersion, saturable absorption, spectral/spatial filtering, etc, have been equivalently integrated into this simulation model with a shorter fiber length, i.e., 50 cm in this case, such that it takes only about 3 hours to finish a single simulation of generating 3D soliton molecules, which is in sharp contrast to that of taking about 3 days to finish a single simulation when using the same fiber length as that of the experiment (i.e., 5-m gain fiber and 4.5-m passive fiber, to be further discussed in the latter part of this section).

The propagation of the 3D light field in the GRIN gain fiber is described by the generalized multimode nonlinear Schrödinger equations (GMMNLSEs)[1], i.e.,

$$\partial_z A_p(t;z) = i\delta\beta_0^{(p)} A_p - \delta\beta_1^{(p)} \partial_t A_p + \sum_{m=2}^{3} i^{m+1} \frac{\beta_m^{(p)}}{m!} \partial_t^m A_p + i\frac{n_2\omega_0}{c} \sum_{l,m,n}^{N} S_{plmn}^K A_l A_m A_n^* , \quad (S1)$$

where, $A_p(t;z)$ is the field envelope of the spatial mode p. $\delta\beta_0^{(p)}$ and $\delta\beta_1^{(p)}$ are the propagation constant and group velocity of the spatial mode p. $\beta_m^{(p)}$ is the m-order dispersion coefficient. n_2, ω_0 and c are the nonlinear refractive index, center angular frequency and speed of light, respectively. S_{plmn}^K is the nonlinear coupling coefficient.

Then, the 3D light field gain of the GRIN gain fiber, as described in ref. 2, can be written as,

$$g(x,y,\omega;z) = \frac{g_0(\omega)}{1 + \int |A(x,y,t;z)|^2 dt / F_{sat}}, \quad (S2)$$

$$\text{with } A(x,y,t;z) = \sum_p \frac{F_p(x,y)A_p(t;z)}{\sqrt{\iint F_p(x,y)dxdy}},$$

where, F_{sat} is the saturation fluence. g_0 is the small signal gain coefficient, and $F_p(x,y)$ is the transverse-mode-field distribution of mode p. The saturable absorption effect is established using a transfer function after the gain fiber propagation, i.e.,

$$A(x,y,t;z) \to A(x,y,t;z)\sqrt{1-(1+|A(x,y,t;z)|^2/I_{sat})^{-1}}, \qquad (S3)$$

where, I_{sat} is the saturation intensity of the absorber, which is set to be 50 GW/cm^2.

The oscillation signal is extracted with a constant ratio, i.e.,

$$A(x,y,t;z) \to A(x,y,t;z)\sqrt{0.4}\,. \qquad (S4)$$

For a moderate calculation time, here only 10 transverse modes are considered without loss of generality.

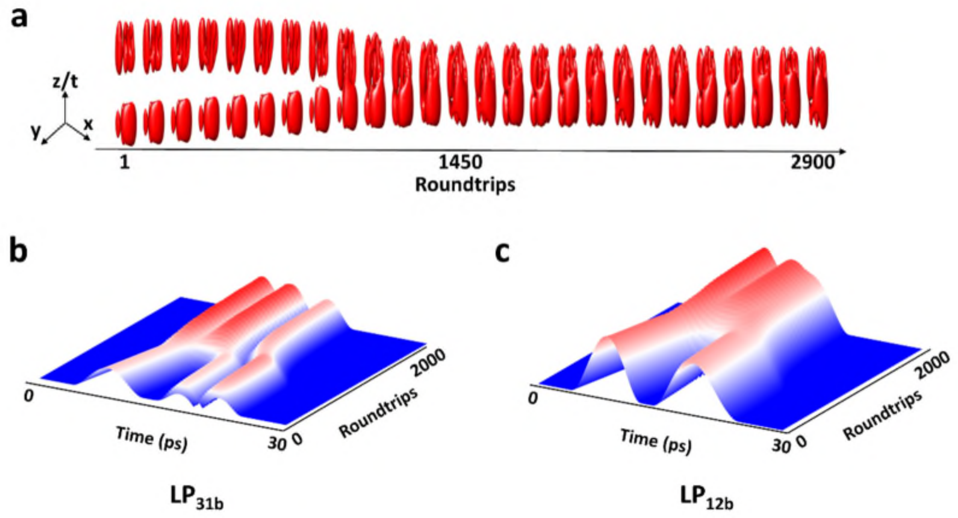

Supplementary Figure 1 | Birth of 3D soliton molecules in the simulation. a. Formation of the soliton molecule. Here, 10 transverse modes are included. **b**. Formation of the triple-soliton molecule in the transverse mode LP_{31b}. **c**. Formation of the dual-soliton molecule in the transverse mode LP_{12b}.

The formation of 3D soliton molecules is presented in **Supplementary Figure 1a**. Here, 10 transverse modes are included in the numerical simulation. Diverse landscapes can

be obtained for different transverse modes. As shown in **Supplementary Figure 1b**, a triple-soliton molecule is generated in the transverse mode LP_{31b}, while a dual-soliton molecule in the transverse mode LP_{12b} (**Supplementary Figure 1c**). The 3D soliton molecule also exhibits internal dynamics after formation, as shown in **Supplementary Figure 2**.

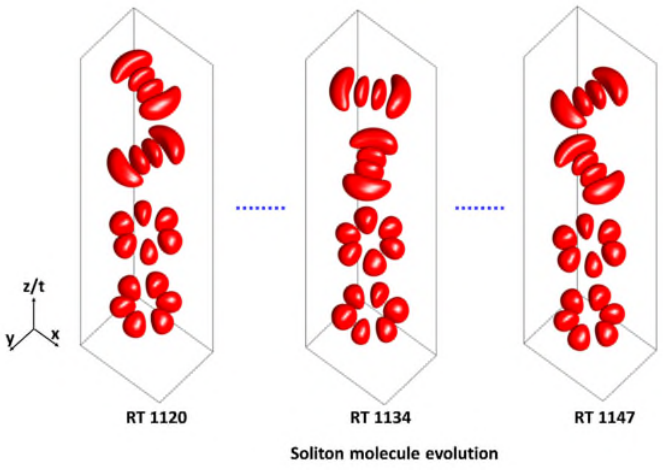

Supplementary Figure 2 | Internal motion of 3D soliton molecules after formation.

Supplementary Figures 3a–d show the temporal evolutions in two different speckle grains (SGs), i.e., observing in different locations of the *x*-*y* plane. The two solitons in **Supplementary Figure 3a** exhibit out-of-step pulsation, displayed as pulsation on the sideband of the autocorrelation (**Supplementary Figure 3c**). The evolution of the relative phase between the two solitons shown in **Supplementary Figure 3e** reveals the period oscillation. In the other speckle grain, however, there coexist four solitons, as shown in **Supplementary Figure 3b**, which exhibits a more complicated temporal evolution landscape, compared to that of **Supplementary Figure 3a**. Complicated autocorrelation evolution and relative phase evolution are also observed, as shown in **Supplementary Figures 3d,f**.

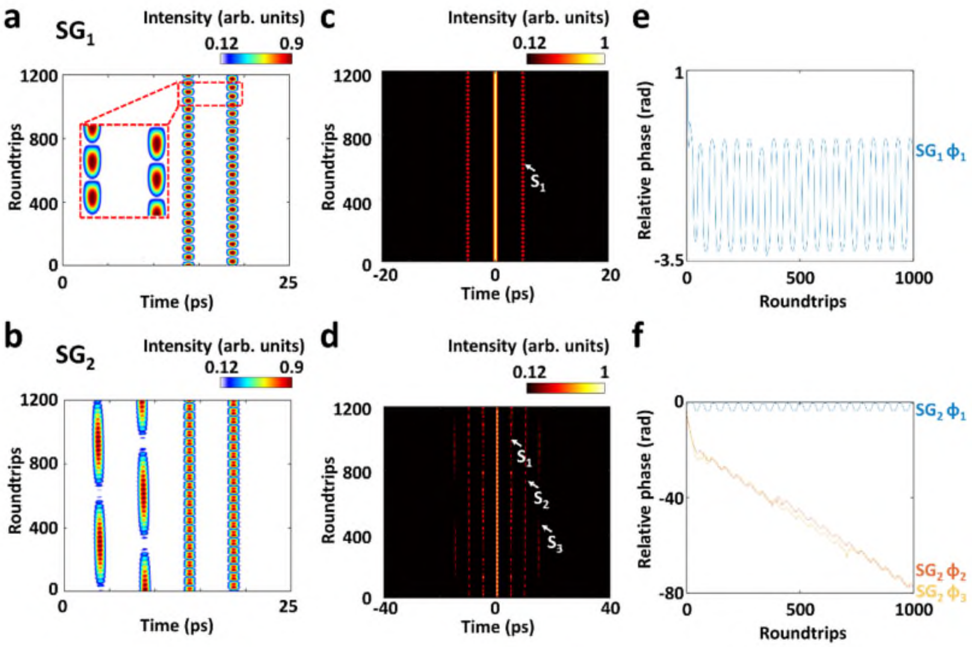

Supplementary Figure 3 | Evolutions of 3D soliton molecules in different speckle grains (SGs). a,b. Temporal evolutions of 3D soliton molecules in two different speckle grains. The inset of **a** shows the close-up of the temporal evolution of 3D soliton molecules. **c,d**. Corresponding autocorrelation evolutions. **e,f**. Relative phase evolutions of **a** and **b** in the time domain.

As illustrated in **Supplementary Figure 4**, the 3D soliton molecule shows diverse evolution landscapes in different speckle grains. When observing in the complete mode area, four stable temporal solitons are generated (**Supplementary Figure 4a**). In contrast, different evolution landscapes are presented when observing in different speckle grains, i.e., **Supplementary Figures 4b–d**. It is worth noting that, although the solitons show diverse intensity evolutions, their temporal separation is consistent throughout the evolution process. In addition, the absent soliton in a speckle grain may present in other speckle grains, implying the possibility of spatiotemporal coupling.

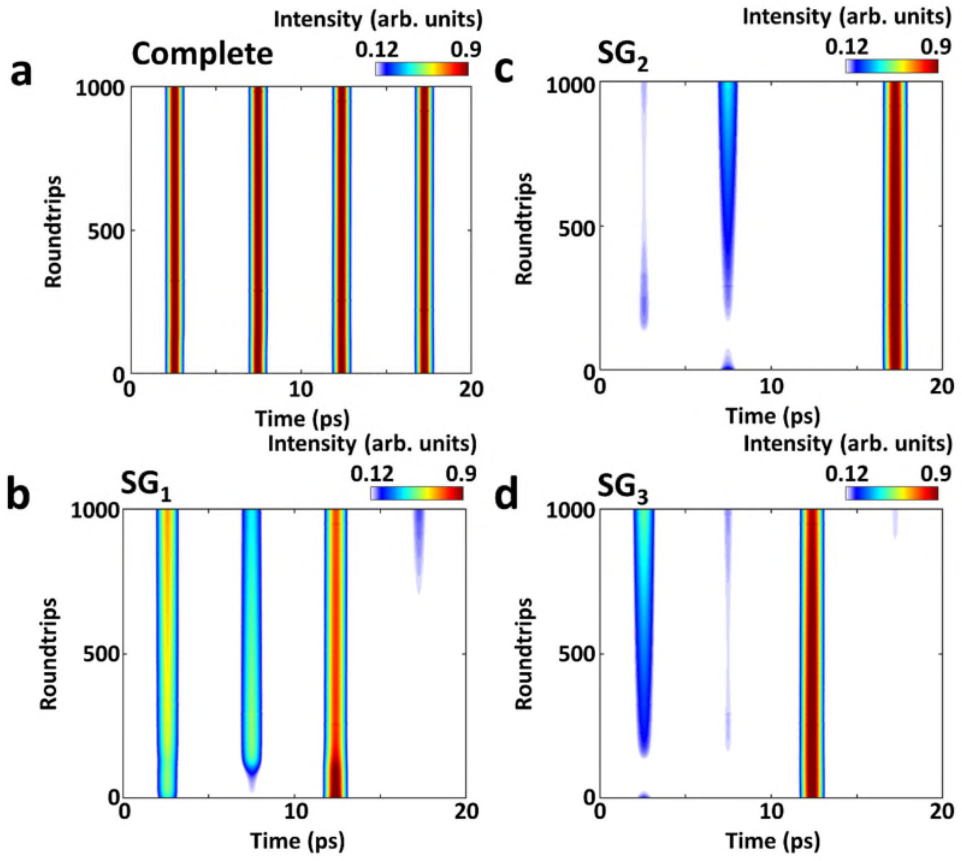

Supplementary Figure 4 | Evolutions of 3D soliton molecules in different speckle grains. a. Temporal evolution of 3D soliton molecules in the complete mode area, i.e., averaging over all the speckle grains. **b**–**d**. Temporal evolutions in three different speckle grains.

As shown in **Supplementary Figure 5**, on the other hand, the 3D dual-soliton molecule shows a clear temporal separation, compared to that of **Fig. 3**. Furthermore, here the two bound solitons manifest out-of-step pulsation evolution when observing in specific speckle grains, e.g., **Supplementary Figures 5c,d**, manifesting a larger modulation depth. In contrast, in the complete mode area the 3D dual-soliton molecule remains stable. It is also noticed that the solitons inside the molecule, as well as the molecules in different speckle grains, exhibit a time delay, as indicated in **Supplementary Figure 5c**.

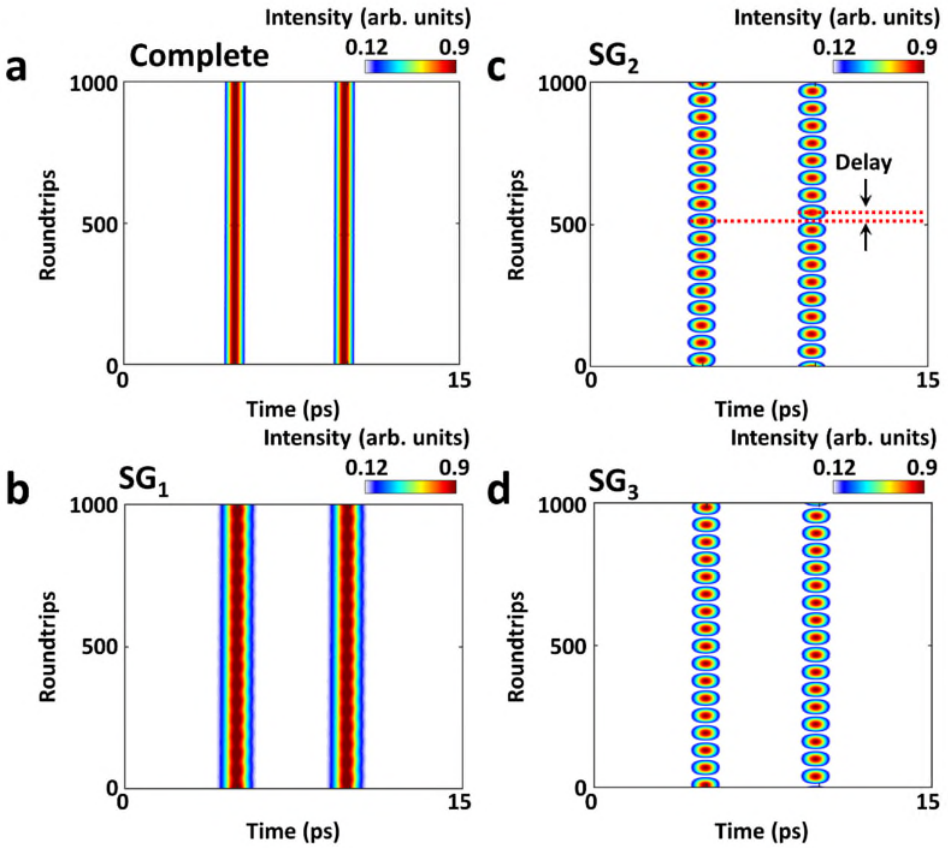

Supplementary Figure 5 | Evolutions of 3D soliton molecules with pulsation characteristics. a. Temporal evolution of 3D soliton molecules in the complete mode area. **b**–**d**. Temporal evolutions in three speckle grains that show different pulsation landscapes.

Supplementary Figure 6 shows another case of 3D soliton molecules that presents different numbers of solitons in different speckle grains. Here, triple-soliton and dual-soliton molecules coexist (**Supplementary Figures 6a,b**). The autocorrelation trace of the dual-soliton molecule (**Supplementary Figure 6f**) evolves similar to that of **Fig. 2f**. In contrast, the three slots of roundtrips of the autocorrelation trace manifest three different landscapes (**Supplementary Figures 6c–e**).

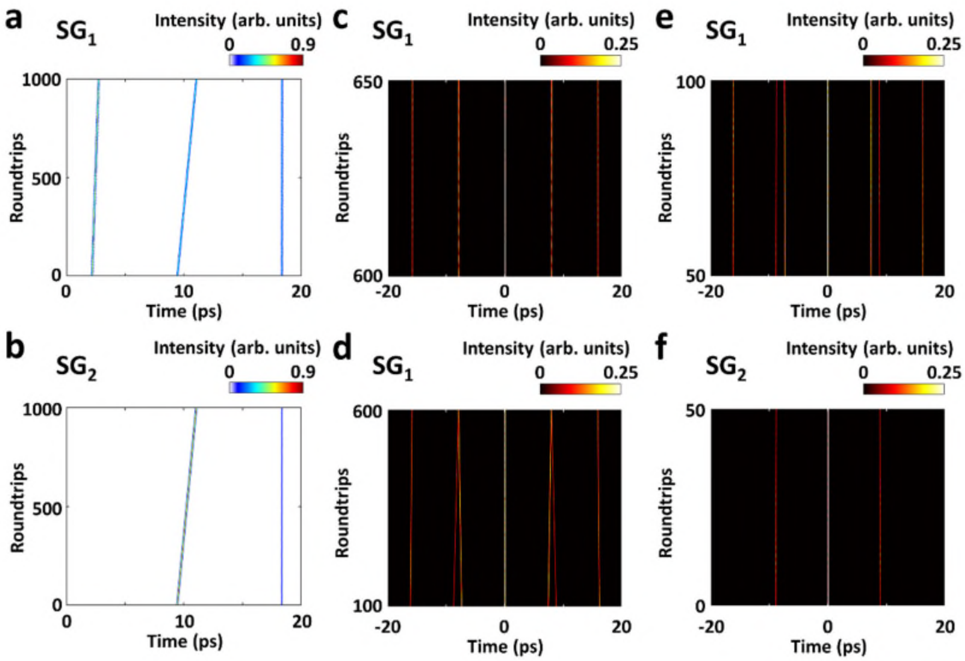

Supplementary Figure 6 | Evolutions of 3D soliton molecules involving different numbers of solitons in different speckle grains. a,b. Temporal evolutions of 3D soliton molecules in two different speckle grains. **c**–**e**. Autocorrelation evolutions of **a** in different slots of roundtrips. **f**. Autocorrelation evolution of **b**.

As mentioned in the beginning of this section, an equivalently shorter fiber is used in the numerical simulation of this work. To identify if this equivalent simulation model using a shorter fiber length can provide good enough qualitative analysis, in contrast to the case using a consistently long fiber (i.e., the same as that of the experiment), we conduct numerical simulation by using a laser cavity the same as that of the experiment, i.e., composed of 5-m gain fiber and 4.5-m passive fiber (noted that, there is also a small part of free space, about 30 cm). As can be observed from **Supplementary Figure 7**, similar dynamics of 3D soliton molecules, like **Supplementary Figure 5**, are successfully generated. In this case, the massive calculation of (3+1)-D light field ($x,y,t;z$) takes about 3 days to finish a single simulation of generating 3D soliton molecules, making a sharp contrast to that of the equivalent simulation model (taking only about 3 hours). Although the use of generalized multimode nonlinear Schrödinger equations (GMMNLSEs) can, to some extent, reduce the computation, it is still time-

consuming for the (3+1)-D simulation of complex laser dynamics over a long evolution time, especially when many transverse modes (several tens to hundreds) are included.

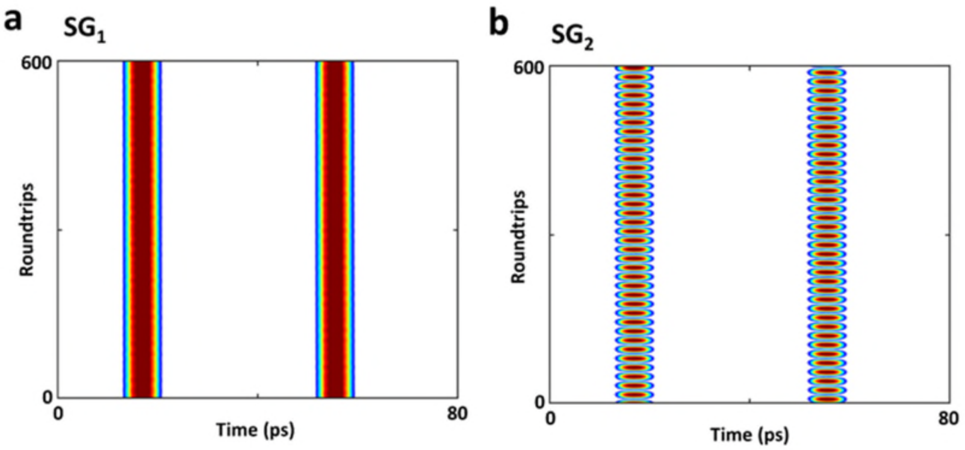

Supplementary Figure 7 | Simulated temporal evolutions of the 3D soliton molecule using a fiber laser cavity the same as that of the experiment. a. Temporal evolution in SG_1. **b**. Temporal evolution in SG_2. Here, the simulation model consists of 5-m gain fiber and 4.5-m passive fiber, which is consistent with that of the experiment.

Supplementary Note 2: Multimode soliton oscillator

The schematic diagram of the spatiotemporal mode-locked (STML) multimode fiber laser used in the work is presented in **Supplementary Figure 8**. An Yb-doped gain fiber (Nufern LMA-YDF-15/130-VIII, 5 m length, 15 μm core diameter) is pumped by a multimode laser diode (~30 W maximum power, 976 nm wavelength) through a signal-pump combiner (SPC) with a multimode fiber pigtail (Nufern LMA-GDF-15/130 0.08/0.46NA, ~2 m length, 15 μm core diameter). A multimode GRIN fiber (Thorlabs GIF625, 2.5 m length, 62.5 μm core diameter) is fusion-spliced to the gain fiber, and a large core offset is applied to excite the higher-order modes. Two collimators (Cols) are utilized to launch the laser beam into free space and couple it back into the fiber. Two half-wave plates (λ/2) and two quarter-wave plates (λ/4) are used for controlling the state of polarization, in conjunction with a narrow bandpass filter (F, Semrock LL01-1064-12.5, ~4 nm bandwidth) and a polarization-dependent isolator (ISO) for realizing the STML operation. A 50:50 beam splitter (BS) is employed to extract the laser signal. The roundtrip time of the laser cavity is about 47.8 ns, corresponding to a fundamental repetition rate of ~20.9 MHz. The right inset of **Supplementary Figure 8** shows a typical mode profile of the STML laser.

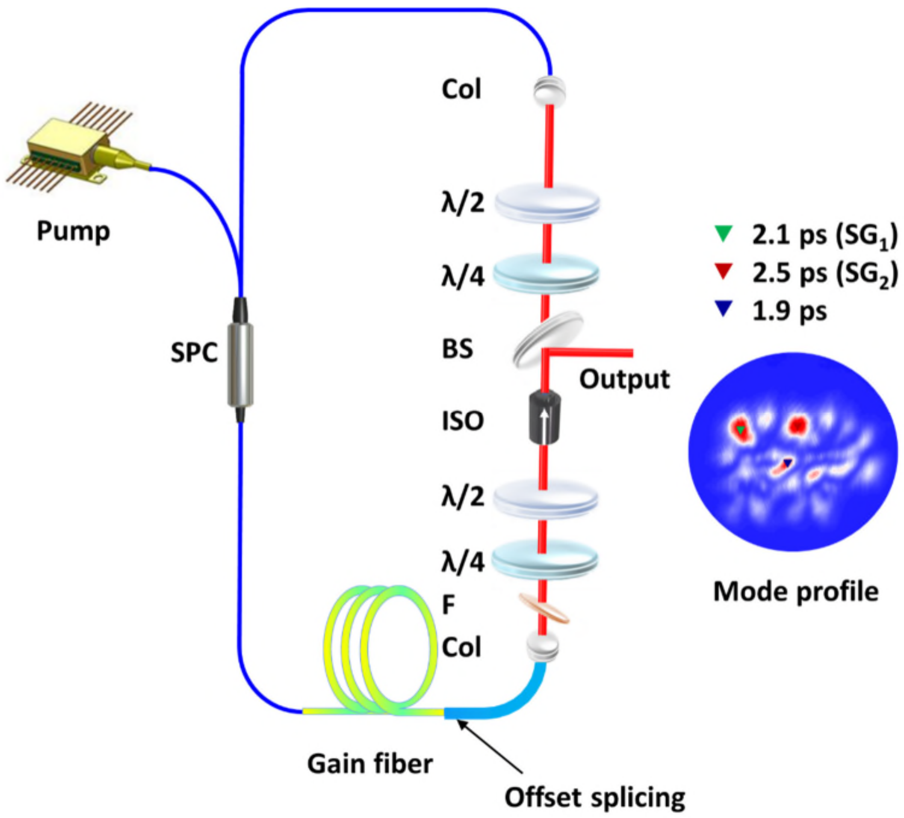

Supplementary Figure 8 | Schematic diagram of the spatiotemporal mode-locked (STML) multimode fiber laser. Right inset shows the specked mode profile, wherein the pulsewidths in three different speckle grains are also provided. BS: beam splitter. Col: collimator. F: filter. ISO: isolator. SPC: signal-pump combiner. λ/2: half-wave plate. λ/4: quarter-wave plate.

Supplementary Note 3: Multispeckle spectral-temporal (MUST) measurement system

3.1. Details of the setup

The schematic diagram of the single-shot MUST measurement system is shown in **Supplementary Figure 9**. The beam size of the STML laser being measured is enlarged by a 5× magnification telescope composed of lenses L_1 and L_2 (30 mm and 150 mm focal lengths, respectively). The magnified laser beam is then launched to the MUST measurement system. In the MUST measurement system, the laser beam is split by two BSs with ratios of 30:70 and 50:50, respectively. The signals of three speckle grains of the multimode laser beam are individually received by three single-mode fiber (SMF) probes. The collected signals propagate through different optical delay lines (ODLs), which are subsequently combined by a 3×2 optical coupler (OC) for optical time division multiplexing (OTDM). The OTDM signal is split into two branches, one of which is directly detected by a high-speed photodiode (PD_1, 12 GHz bandwidth). The other branch is launched to a long SMF (Nufern 1060-XP, 8 km length), wherein the optical signal propagates back and forth in the SMF, providing a large group delay dispersion (GDD, about -0.6 ns/nm in this case) for real-time spectroscopy. The time-stretched signal is detected by another high-speed PD (PD_2). The outputs of the PDs are finally recorded by a multi-channel real-time oscilloscope at a sampling rate of 80 GS/s.

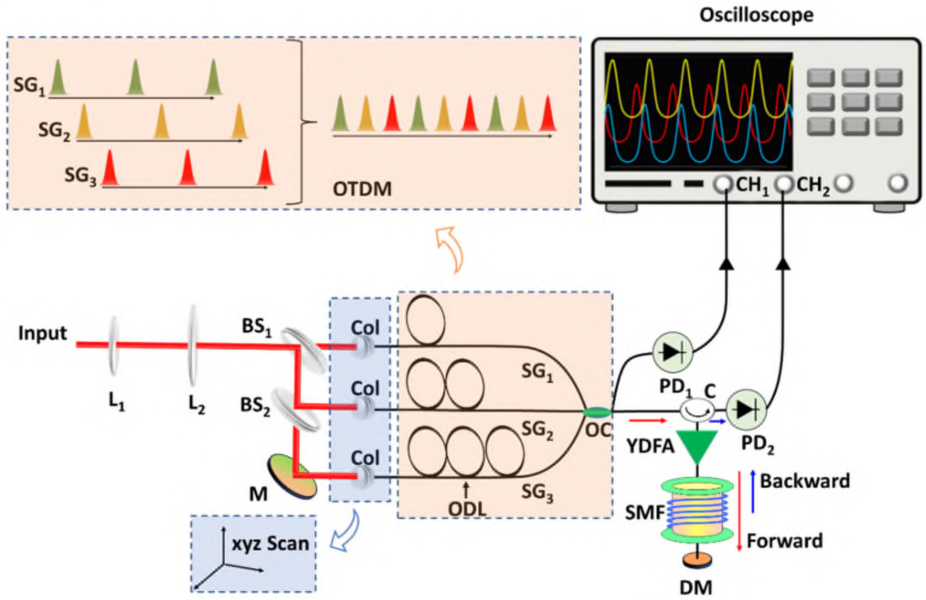

Supplementary Figure 9 | Configuration of the MUST measurement system. BS: beam splitter. C: circulator. Col: collimator. L: lens. M: mirror. DM: dielectric mirror. OC: optical coupler. ODL: optical delay line. OTDM: optical time division multiplexing. PD: photodiode. SMF: single-mode fiber. SG: speckle grain. YDFA: Yb-doped fiber amplifier.

To avoid the potential spectral distortion from using a YDFA in the dispersive Fourier transformation (DFT), we have carefully optimized the parameters of the YDFA, mainly adjusting the length of the Yb-doped gain fiber used in the YDFA to suppress the nonlinear effects, which potentially occur on the forward direction. It is also worth noting that, the YDFA imparts limited change to the spectral shape of the amplified signal on the backward direction, which is because the pulse signal has been largely broadened after double-passing the long SMF that is highly dispersive, such that its peak power is sufficiently low and the nonlinear effects are prevented.

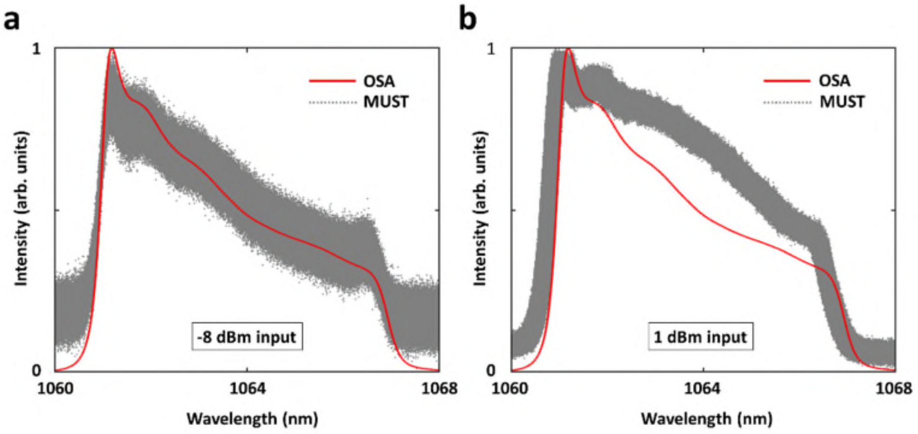

Supplementary Figure 10 | Optical spectra collected by the standard optical spectrum analyzer (OSA) and MUST measurement system that involves a YDFA. a,b. Spectroscopy measurements with different input signal powers, i.e., -8 dBm (**a**) and 1 dBm (**b**), respectively. Here, the net gain is fixed to be ~20 dB for both cases. The OSA curves were recorded before the YDFA, while the MUST curves were recorded after the YDFA and double-pass SMF units, i.e., right before the PD_2.

We tested the spectroscopy performance of the MUST measurement system employing the YDFA, and the results are shown in **Supplementary Figure 10**. In this case, a single-mode mode-locked (SM ML) laser with a repetition rate comparable to that of the STML laser utilized in the manuscript. In each measurement, we fixed the net gain of the YDFA to be ~20 dB, which is comparable to that of the loss of double-passing the long SMF (about 16 dB). Two different power levels of the input signal were tested, i.e., -8 dBm and 1 dBm, respectively. The results show that, the optical spectrum of the amplified signal is well maintained for a low signal power level, i.e., the case of -8 dBm, while it could be slightly changed for a higher signal power level, i.e., 1 dBm. In this work, the power of the speckle-resolved signal captured by the MUST measurement system is typically less than -10 dBm, such that the accuracy of the spectroscopy measurement can be ensured.

3.2. Spatial resolution of the MUST measurement system

For collecting the spectral-temporal signal from a single speckle grain, a single-mode fiber that can serve as spatial filter is useful. To do so, we utilize three probes to collect

the spectral-temporal signals of three different speckle grains, and each probe consists of a single-mode fiber and a collimator for light coupling (**Supplementary Figure 11**). To freely access different speckle grains, the collimator is mounted on the translation stage. It is worth noting that, each bright speckle grain is a coherent spot resulted from the constructive interference. In this sense, a spatial resolution that can resolve the speckle grains is good enough for the MUST measurement, as shown in **Supplementary Figure 12**. To ensure this capability, the collimator is associated with a telescope for magnifying the size of the speckle grains, and such a configuration can provide a spatial resolution of about 2.6 μm

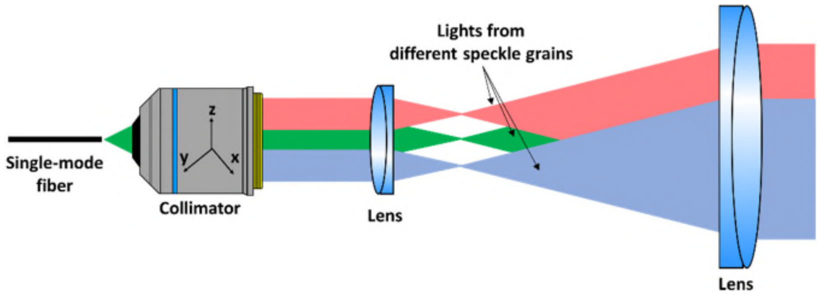

Supplementary Figure 11 | Configuration of collecting lights from different speckle grains using single-mode fibers. Here, the collimator is mounted on the translation stage.

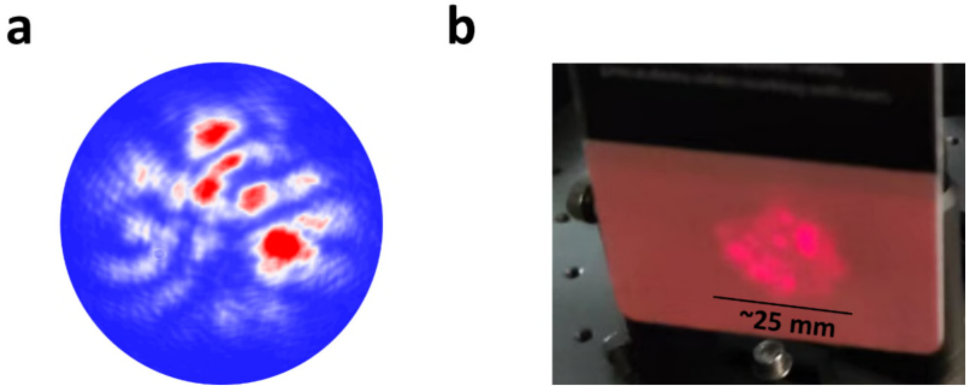

Supplementary Figure 12 | Speckle grains of the STML multimode fiber laser. a. Beam profile captured by a CCD camera. **b**. Beam profile shined on a photosensitive card.

3.3. Channel scaling of the MUST measurement system

To demonstrate the ability of simultaneously measuring more speckle grains, we implement a MUST measurement system with six channels, and the spectral evolutions of six different speckle grains are simultaneously captured, as shown in **Supplementary Figure 13**. This implies that, the channel scaling is feasible by using free-space optics, if compactness compromise is not a concern. It should also be pointed out that, here the real-time DFT spectroscopy is performed in a single long-SMF unit that is associated with the OTDM technology. Thus, temporal overlapping of the DFT signal will limit the number of channels, i.e., about 8 channels for the configuration used in this work. To solve this problem, parallel detection with more DFT units and digitizers is helpful. In addition, using micro-lens array and multicore dispersive fiber, as shown in **Supplementary Figure 14**, can potentially further increase the number of channels, but making the measurement system more complicated and expensive. Based on the above discussion, we implemented a MUST measurement system with only three channels in this study, given that resolving the spectral-temporal signals for three speckle grains are already useful for probing the spatial-spectral-temporal dynamics of the 3D soliton molecule, from the perspective of qualitative analysis.

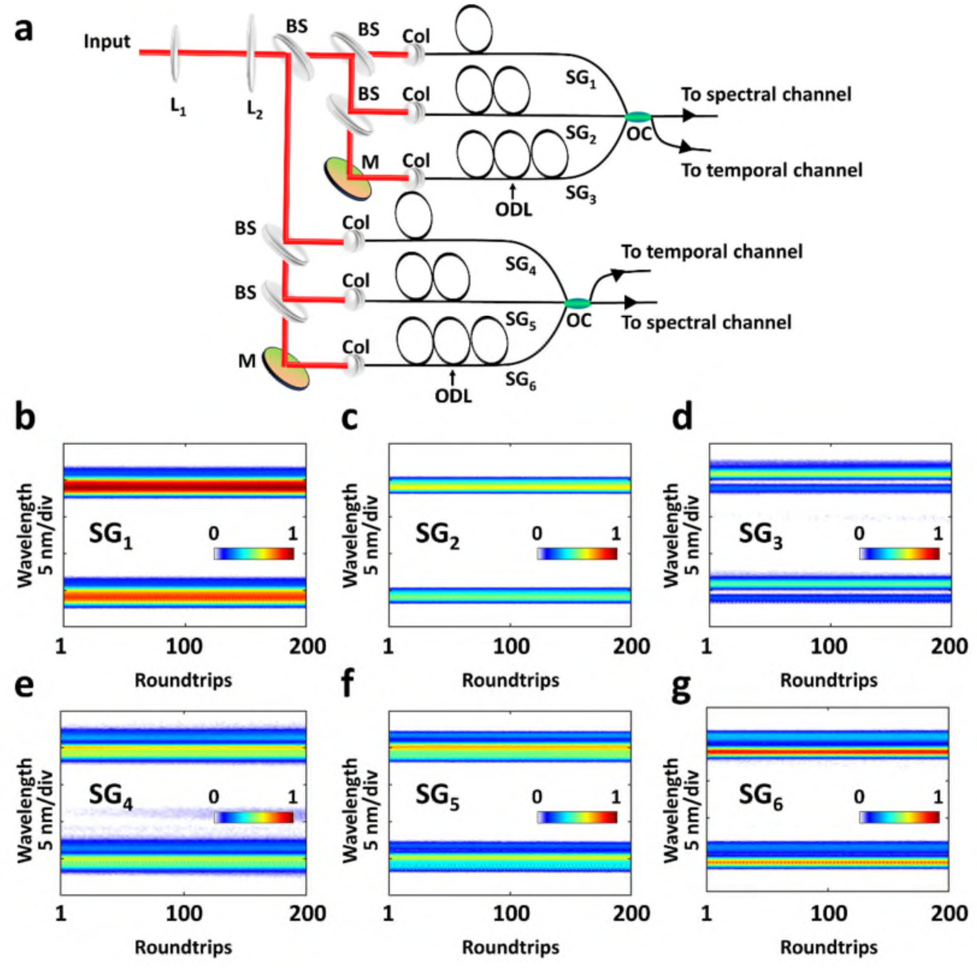

Supplementary Figure 13 | MUST measurement system with six channels. a. Schematic diagram. BS: beam splitter. C: circulator. Col: collimator. L: lens. M: mirror. OC: optical coupler. ODL: optical delay line. SG: speckle grain. **b-g**. Spectral evolutions of six different speckle grains captured by the six-channel MUST measurement system.

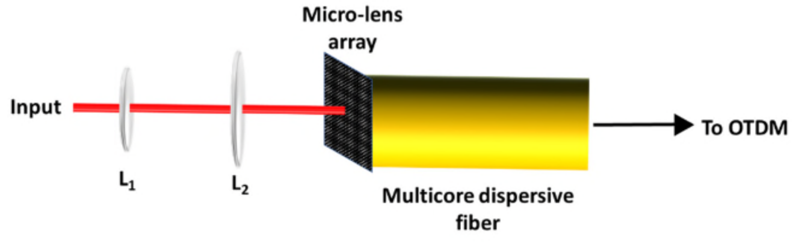

Supplementary Figure 14 | Potential scheme for speckle-resolved optical collection using micro-lens array and multicore dispersive fiber. L: lens. OTDM: optical time division multiplexing.

Supplementary Note 4: Calculation of the field autocorrelation and relative phase extracted from the spectroscopy results

4.1. Calculation procedure and its validity

The flow diagram of the field autocorrelation (FAC) extraction is shown in **Supplementary Figure 15**. Considering a doublet shape of soliton envelops $E_1(t)$ and $E_2(t)$ that have a temporal separation of τ and relative phase of ϕ, we have

$$E(t) = E_1(t) + E_2(t-\tau)e^{-i\phi}. \quad (S5)$$

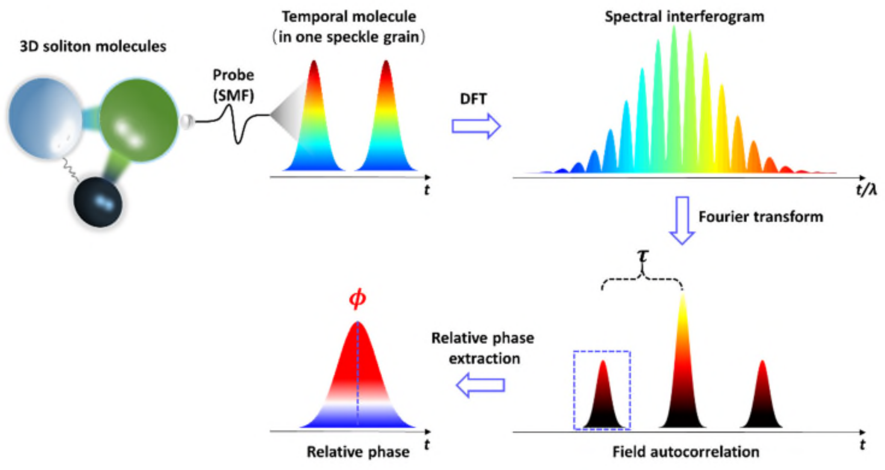

Supplementary Figure 15 | Flow diagram of the field autocorrelation extraction. DFT: dispersive Fourier transformation.

Then, the spectral intensity $\tilde{I}(\omega)$ from the DFT becomes

$$\tilde{I}(\omega) = \left|\tilde{E}(\omega)\right|^2$$

$$= \left|\tilde{E}_1(\omega)\right|^2 + \left|\tilde{E}_2(\omega)\right|^2 + \tilde{E}_1(\omega)\tilde{E}_2^*(\omega)e^{i\omega\tau+i\phi} + \tilde{E}_1^*(\omega)\tilde{E}_2(\omega)e^{-i\omega\tau-i\phi}, \quad (S6)$$

where, ~ represents the result of the Fourier transform (FT) operation. $\tilde{E}(\omega)$, $\tilde{E}_1(\omega)$ and $\tilde{E}_2(\omega)$ are the FT of $E(t)$, $E_1(t)$ and $E_2(t)$, respectively.

For convenience, Eq. ($S6$) can be further rewritten as

$$\tilde{I}(\omega) = \tilde{e}_0(\omega) + \tilde{e}_1(\omega)e^{i\omega\tau+i\phi} + \tilde{e}_{-1}(\omega)e^{-i\omega\tau-i\phi}, \quad (S7)$$

where

$$\tilde{e}_0(\omega) = \left|\tilde{E}_1(\omega)\right|^2 + \left|\tilde{E}_2(\omega)\right|^2,$$

$$\tilde{e}_1(\omega) = \tilde{E}_1(\omega)\tilde{E}_2^*(\omega),$$

and $\tilde{e}_{-1}(\omega)$ represents the complex conjugate of $\tilde{e}_1(\omega)$.

According to the Wiener-Khinchin theorem[3], the FAC function $R(t)$ is given as

$$R(t) = \mathcal{F}^{-1}[I(\omega)]$$

$$= e_0(t) + e_1(t+\tau)e^{i\phi} + e_{-1}(t-\tau)e^{-i\phi}. \qquad (S8)$$

The soliton separation τ can be extracted by measuring the distance between the sidelobes and the center of $R(t)$. To extract the relative phase ϕ, $R(t)$ is filtered by $H(t+\tau)$, which is the time gating function at $t = -\tau$, then we have

$$\phi = \sphericalangle\mathcal{F}[R(\tau)H(t+\tau)]|_{\omega=0} = \sphericalangle\mathcal{F}\left[e_1(t+\tau)e^{i\phi}\right]\Big|_{\omega=0}, \qquad (S9)$$

where, $\mathcal{F}$ denotes the FT operation.

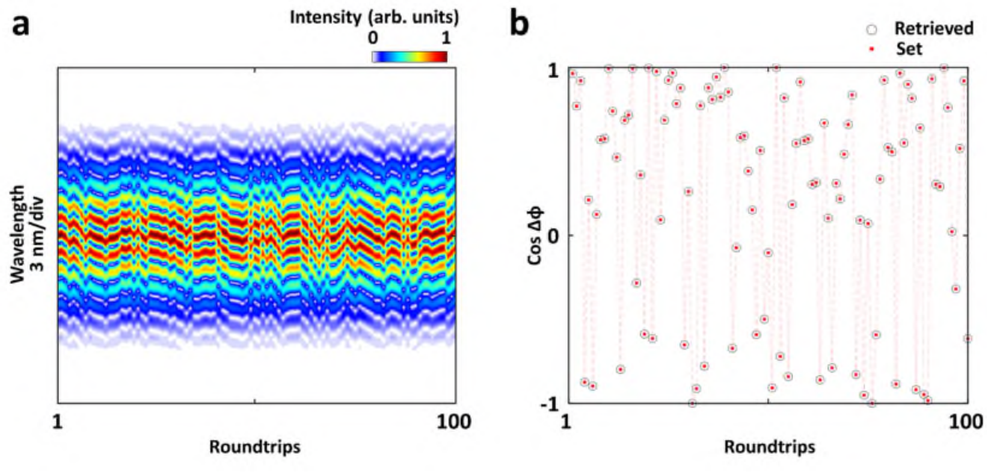

Supplementary Figure 16 | Numerical validation of the phase-retrieval method. a. Interferogram of soliton molecules with preset relative phases. **b**. Retrieved relative phases (black circles) and the preset values (red dots).

To address the reviewer's concern on the validation of the phase-retrieval method used in this work, we numerically create interferograms of soliton molecules with preset relative phases ϕ, as shown in **Supplementary Figure 16a**, and subsequently evaluate the accuracy of the retrieved relative phases using these preset values, as shown in **Supplementary Figure 16b**. As can be observed, a good agreement is obtained, theoretically confirming the feasibility of the phase-retrieval method.

4.2. Accuracy of the phase-retrieval method

In the experimental implementation, the limited bandwidth of the detection system (mainly including the photodetector and digitizer) can impose phase error $\delta\phi$ on the retrieved phase[4], which is given as,

$$\delta\phi \sim \delta\omega_{res}\tau \qquad (S10)$$

with temporal separation τ and spectral resolution $\delta\omega_{res}$ that is written as[5]

$$\delta\omega_{res} = \frac{0.35}{D_2 f_{det}}. \qquad (S11)$$

where, f_{det} is the bandwidth of the optical detection system, i.e., 21 GHz in this case (defined by the digitizer). The phase ambiguity in this work is comparable with that of prior works (**Supplementary Table 1**).

Supplementary Table 1. **Phase ambiguity of real-time spectral interferometry**

Temporal separation τ (ps)	D_2 (ps^2)	Bandwidth f_{det} (GHz)	Phase error $\delta\phi/2\pi$	Ref.
<0.6	18.8	~8	<22.2%	*Science, 356, 50 (2017)*
~2 ps	163	~6	~11.4%	*PRL, 118, 243901 (2017)*
40 ps	178	45	27.8%	*OE, 29, 16362 (2021)*
10 ps	235	20	11.9%	*OE, 30, 21931 (2022)*
**~30 ps**	**360**	**21**	**~22%**	**This work**

To further identify the feasibility of the phase evaluation, we calculate and simulate the roundtrip-evolved retrieved phase for **Fig. 6**. As shown in **Supplementary Figure 17a,b**, the numerical simulation can well reproduce varying features of ϕ_{12}, ϕ_{23}, and ϕ_{13}, e.g., the jump and plateau over the phase evolution (as indicated by the arrows). As marked by the black dashed square in **Supplementary Figure 17a**, a non-trivial fluctuation of ϕ_{12} is observed, also see the close-up shown in **Supplementary Figure 17c**.

From the above discussion, we can conclude that: **(i)** the phase-retrieval method is numerically validated; **(ii)** the bandwidth limitation of the detection system can impart an acceptable phase ambiguity for qualitative study, wherein the major feature of the phase evolution can be retrieved.

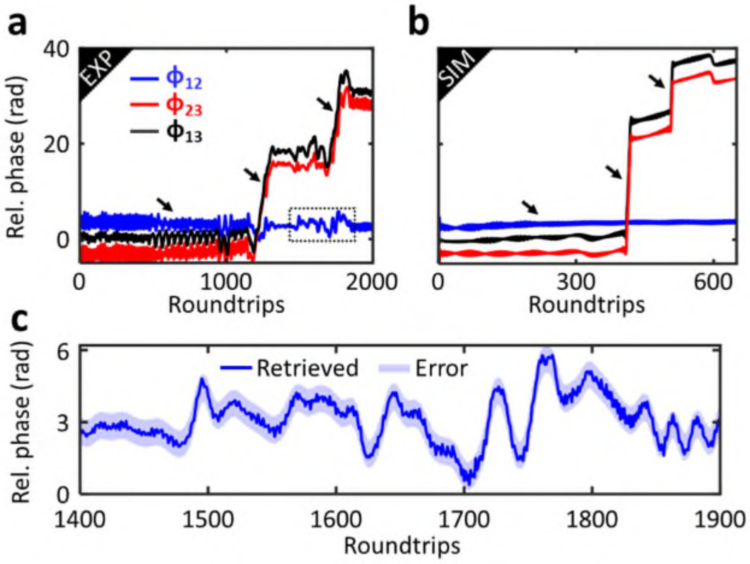

Supplementary Figure 17 | Evolutions of the retrieved phase. a,b. Evolutions of the relative phase retrieved from the experimental (**a**) and simulated (**b**) interferograms. Arrows indicate the similar features shown in the experiment and simulation. **c**. Close-up of ϕ_{12}, indicated by the black dashed square in **a**.

Supplementary Note 5: Birth and internal motion of 3D soliton molecules in the experiment

Supplementary Figure 18 shows the birth dynamics of the 3D soliton molecule measured by the MUST measurement system. Here, only the spectral evolution of SG_1 is presented (**Supplementary Figure 18a**). The corresponding FAC evolution shows that the FAC sidelobes are gradually stabilized after about 1500 roundtrips, as shown in **Supplementary Figure 18b**. In the experimental observation, slow interaction inside the 3D soliton molecule is also captured (**Supplementary Figure 19**).

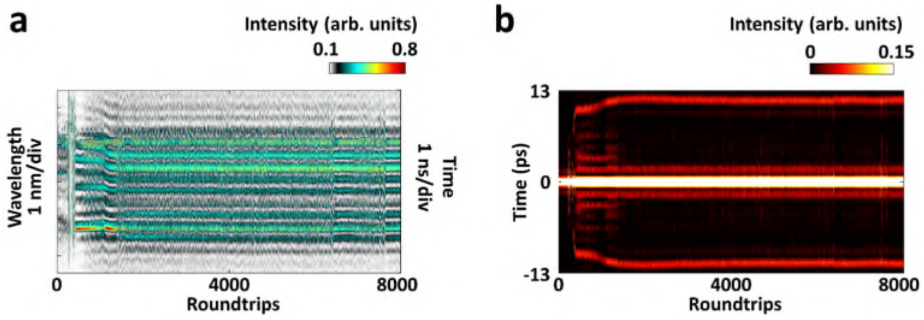

Supplementary Figure 18 | Birth of the 3D soliton molecule in the experiment. a. Spectral evolution during the birth of the 3D soliton molecule, measured by the MUST measurement system. **b**. Corresponding field autocorrelation (FAC) evolution of **a**.

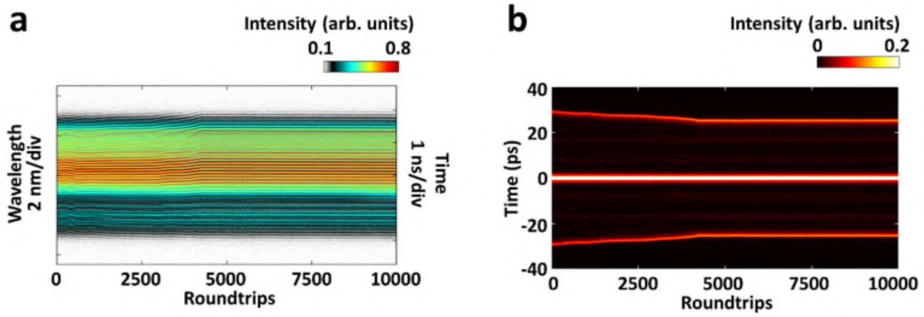

Supplementary Figure 19 | Slow interaction inside the 3D soliton molecule. a. Spectral evolution of the 3D soliton molecule. **b**. Corresponding FAC evolution of **a**. Here, two solitons gradually approach each other.

Supplementary Note 6: Extended figures of 3D soliton molecular dynamics

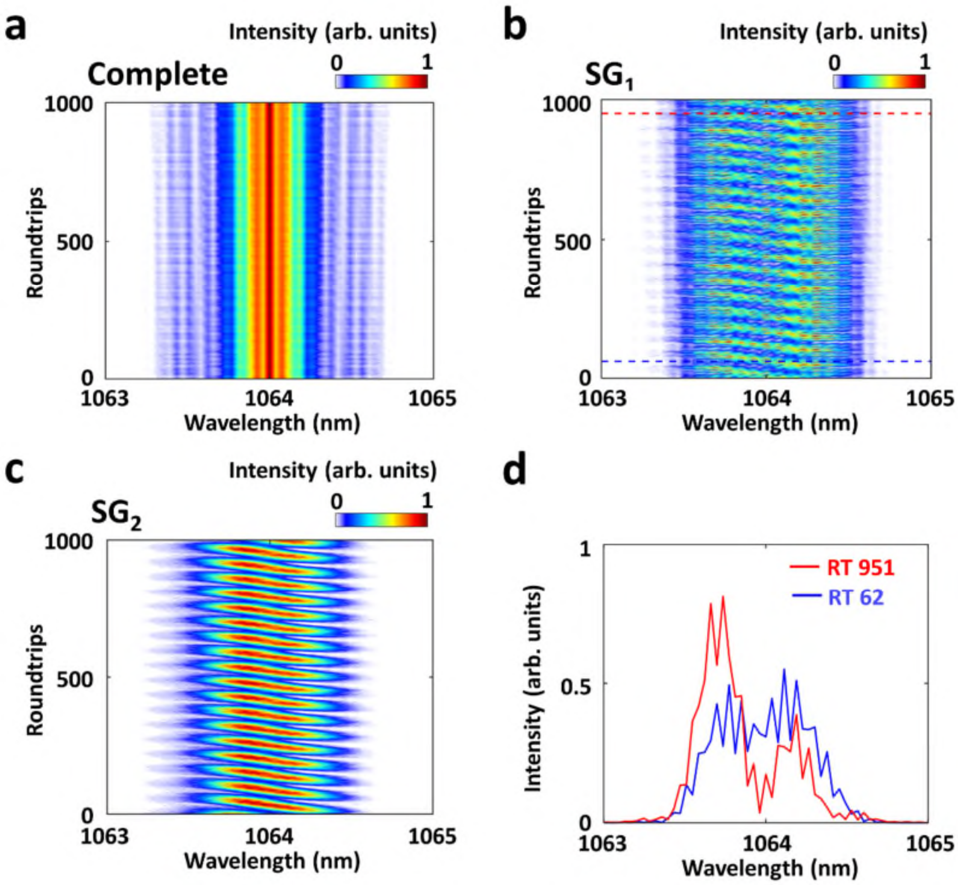

Supplementary Figure 20 | Spectral evolution of the dual-soliton molecule in the numerical simulation. a. Spectral evolution of the dual-soliton molecule in the complete mode area. **b,c**. Spectral evolutions in two different speckle grains (i.e., SG_1 and SG_2). **d**. Spectral intensity profiles of roundtrips 62 and 951 in SG_1, as indicated in **b**.

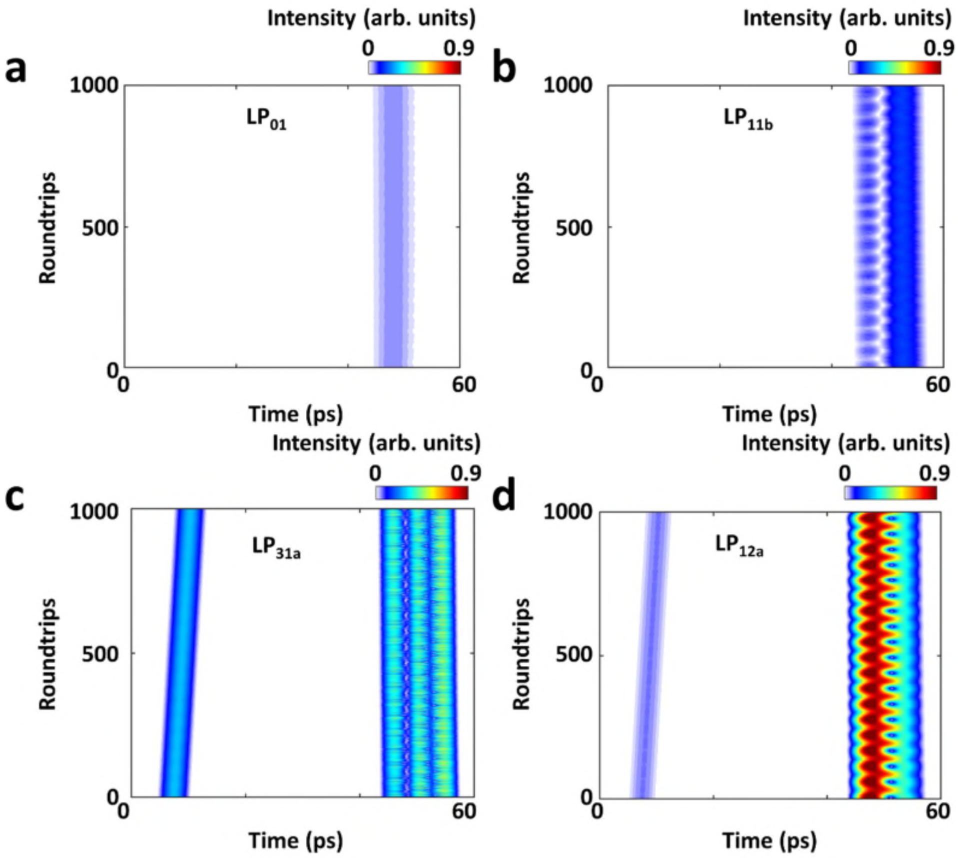

Supplementary Figure 21 | Temporal evolutions of the 3D soliton molecule in different transverse modes. **a**. Temporal evolution of a single 3D soliton in LP_{01}. **b**. Temporal evolution of a dual-soliton molecule in LP_{11b}. Here, it exhibits temporal pulsation characteristics. **c**. Temporal evolution of a triple-soliton molecule accompanied by a drifting soliton in LP_{31a}. **d**. Temporal evolution of a dual-soliton molecule accompanied by a drifting soliton in LP_{12a}.

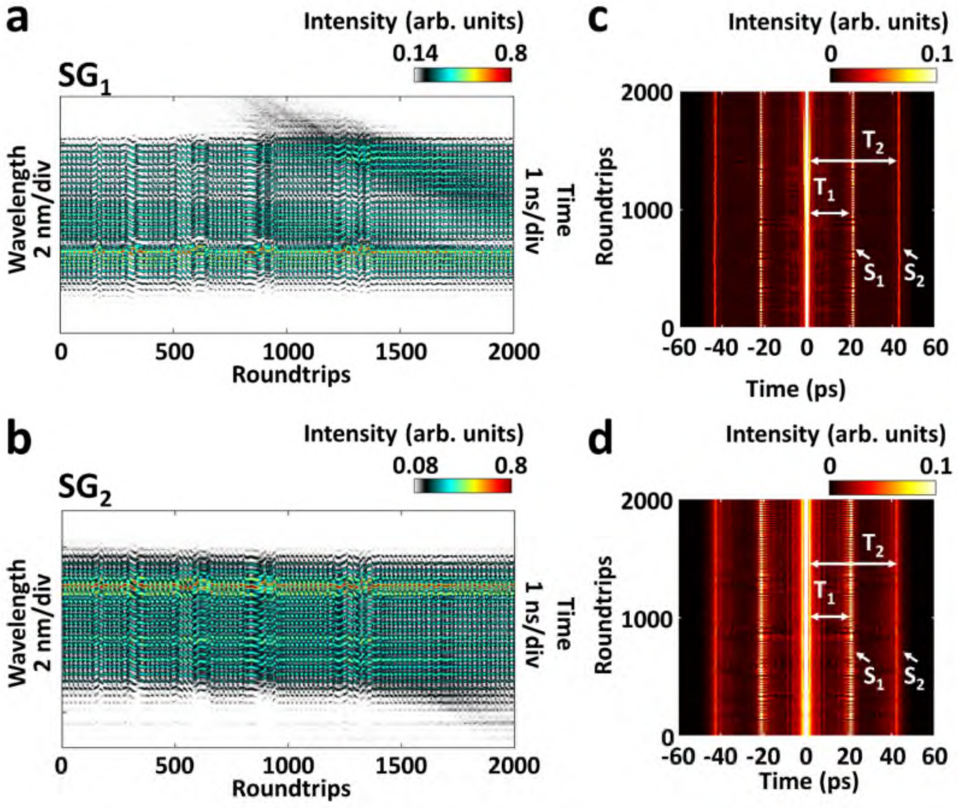

Supplementary Figure 22 | Complicated spectral-temporal dynamics of 3D triple-soliton molecules of Fig. 5. a,b. Complicated spectral evolutions in SG_1 and SG_2. **c,d**. Corresponding FAC evolutions of **a** and **b**.

Supplementary Note 7: Underlying dynamic rules of these observations and profound understandings of these interesting views of 3D soliton dynamics

7.1. Photonic-chemical analogy

To provide an intuitive picture of the underlying dynamic rules of spatiotemporal (ST) soliton molecules, we make a closely-related analogy between the photonic and chemical concepts, as outlined in **Supplementary Figure 23**, and then try to understand each observation using such a photonic-chemical analogy. To do so, the first 10 linearly-polarized (LP) transverse modes, which are defined by the value of the effective refractive index, are employed and marked as multimode elements A-F (top panel of **Supplementary Figure 23)**. The degenerate transverse modes that share an identical propagation constant are regarded as photonic isotopes (denoted as ^{a}X and ^{b}X for multimode element X), wherein the different phases of azimuthal modal components (e.g., 0 or π/2) can mimic the variable neutron number.

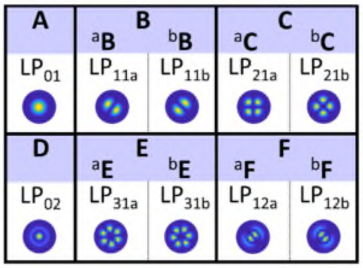

Notation	Chemical analogy	Photonic concept
X (A-F)	Element	LP modes
^{a}X, ^{b}X	Isotopes	Degenerate modes
X_2	Homonuclear diatomic molecule	Soliton molecule with a (degenerate) mode(s)
XY	Heteronuclear diatomic molecule	Soliton molecule with distinguishing modes
X—Y	Bond strength	Average-chirp gradient

Supplementary Figure 23 | Multimode elements and concepts of the photonic-chemical analogy.

7.2. Typical soliton molecules and their chemical analogues

Here we discuss two different 3D soliton molecules, i.e., 3D dual-soliton and triple-soliton molecules, and try to understand them from the chemical perspective.

3D dual-soliton molecule

To understand these observed dynamics from the perspective of photonic-chemical analogy discussed above, here we discuss two typical kinds of 3D dual-soliton molecules (**Supplementary Figure 24**): the first kind is made up of two different linearly-polarized (LP) modes (denoted as kind I), and the second kind is constituted by a (degenerate) mode (denoted as kind II).

For kind-I dual-soliton molecules, the pulses in two distinguishing transverse modes initially propagate at different group velocities due to the modal dispersion of the multimode fiber. Once they overlap in the time domain, it gives rise to the spatiotemporal interaction though the interplay of IM-XPM and IM-FWM[6-8], as shown in **Supplementary Figure 24a**. This process can be described by the generalized nonlinear Schrödinger equation i.e.,

$$\frac{\partial A_p}{\partial z} = -i\frac{\beta_{21}}{2}\frac{\partial^2 A_p}{\partial t^2} + i\gamma_{pp}\left|A_p\right|^2 A_p + \underbrace{2i\gamma_{pq}\left|A_p\right|^2 A_q}_{IM-XPM} + \underbrace{i\gamma_{pq}{A_p}^2{A_q}^* exp(-2i\Delta\beta z)}_{IM-FWM}, \quad (S12)$$

with $\gamma_{pp} = \frac{n_2\langle\omega_1\rangle Area_p}{c}, \gamma_{pq} = \frac{n_2\langle\omega_1\rangle}{c}\frac{\iint_{-\infty}^{\infty}\left|F_p(x,y)\right|^2\left|F_q(x,y)\right|^2 dxdy}{\iint_{-\infty}^{\infty}\left|F_p(x,y)\right|^2 dxdy \iint_{-\infty}^{\infty}\left|F_q(x,y)\right|^2 dxdy},$

where $A_p(t;z)$ and $A_q(t;z)$ are the field envelopes of the LP_{21a} and LP_{31a} modes, respectively; while $F_p(x,y)$ and $F_q(x,y)$ are the corresponding transverse-mode-field distributions. $Area_p$ and β_{21} are the effective mode area and second-order dispersion of the LP_{21a} mode, respectively. n_2 is the nonlinear refractive index. $\Delta\beta$ represents the propagation constant mismatch, and c is the speed of light. To intuitively understand the mechanism of providing the binding force, we try to interpret it through an analogous 'chemical bond' generated between two distinguishable atoms

(e.g., ^{a}C for LP_{21a} and ^{a}E for LP_{31a} as indicated in **Supplementary Figure 24a**) by means of the Gordon-Mollenauer approach[9], and the force can be expressed as the 1D gradient of the average chirp $\langle\omega_1\rangle$ (also see **Methods**), i.e.,

$$f = \frac{d\langle\omega_1\rangle}{dz} = \frac{i}{2W}\int_{-\infty}^{+\infty} dt\left[\frac{d}{dz}\left(A_p\partial_t A_p{}^* - A_p{}^*\partial_t A_p\right)\right], \quad W = \int_{-\infty}^{+\infty}\left|A_p\right|^2 dt. \quad (S13)$$

By combining it with the equation of $\partial A_p/\partial z$ mentioned before, we have

$$f = \frac{\gamma_{pq}}{W}\int_{-\infty}^{+\infty} dt\{Re\left(A_p\partial_t A_p{}^*\right)Re\left[A_p{}^*A_q\left(t-\delta_{pq}z;z\right)\times\left(2+e^{2i\Delta\beta z}\right)\right]$$

$$-Im\left(A_p\partial_t A_p{}^*\right)Im\left[A_p{}^*A_q\left(t-\delta_{pq}z;z\right)\times\left(3e^{2i\Delta\beta z}-2\right)\right]\}, \quad (S14)$$

with $\delta_{pq} = \beta_{11}(\langle\omega_1\rangle) - \beta_{12}(\langle\omega_2\rangle) + \beta_{21}\times\langle\omega_1\rangle - \beta_{22}\times\langle\omega_2\rangle$,

where δ_{pq} accounts for the group velocity mismatch between LP_{21a} and LP_{31a} modes. β_{11} and β_{12} are the first-order dispersions of LP_{21a} and LP_{31a} modes, respectively. β_{22} and $\langle\omega_2\rangle$ are the second-order dispersion and average chirp of LP_{31a} mode. The calculated results are shown in **Supplementary Figure 24b**, wherein the binding force reaches its maximum $f = 4.3\times10^{-4}$ in the reaction (as marked by the arrow in **Supplementary Figure 24b**), resulting in the assembling of molecule 'CE'. It is noteworthy that this 3D dual-soliton molecule, as a photonic counterpart of heteronuclear diatomic molecule, is only accessible in the ST mode-locking that involves diverse multimode elements.

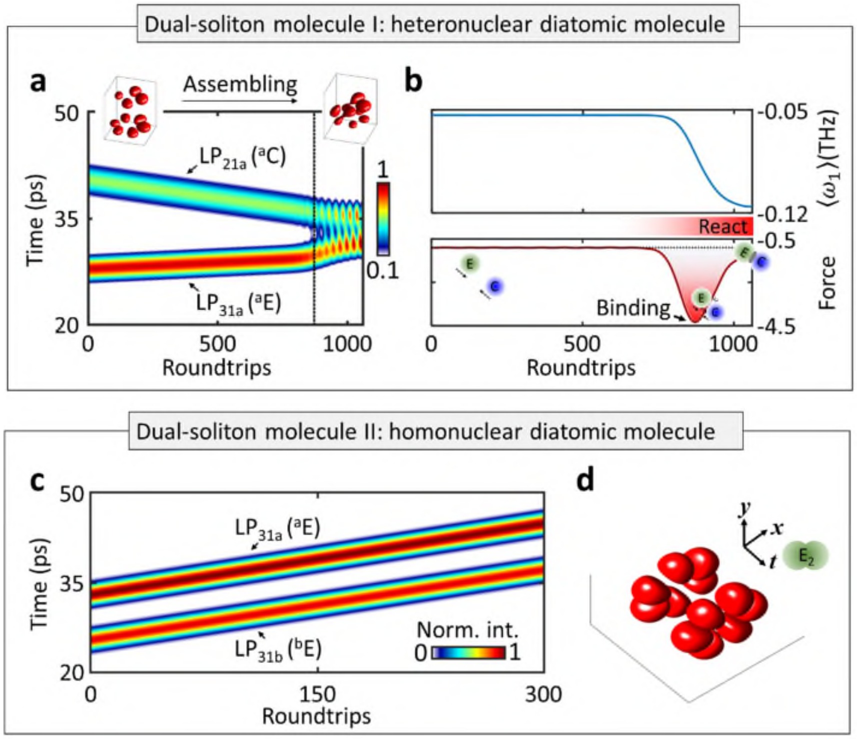

Supplementary Figure 24 | Temporal dynamics of two different 3D dual-soliton molecules. a,b. Temporal evolution (**a**), corresponding variations of average chirp $\langle \omega_1 \rangle$ (top panel of **b**) and binding force (bottom panel of **b**) for the LP_{21a} mode in the case that a dual-soliton molecule in analogy with heteronuclear diatomic molecule is produced. Here, a negative value represents an attraction force, while a repulsion force for a positive value. Insets of **a** illustrate the isosurface plots of the 3D dual-soliton molecule before and after the molecular assembling. **c,d**. Temporal evolution and 3D view of another dual-soliton molecule in analogy with homonuclear diatomic molecule.

For kind-II dual-soliton molecules, the pulses in a pair of degenerate modes (e.g., LP_{31a} and LP_{31b} as indicated in **Supplementary Figures 24c**) have very similar characteristics, particularly their propagation constant, and they evolve in a landscape akin to these 1D soliton molecules, while their synchronization can be further reinforced by the maximum gain principle in the ST mode-locking[2,10]. In contrast to the case of kind I, kind-II 3D soliton molecules resemble homonuclear diatomic molecules. Despite its similarity with these 1D counterparts, the binding mechanism driven by the modal degeneracy and maximum gain principle still has no analogue in the scenario of single-mode mode-locking.

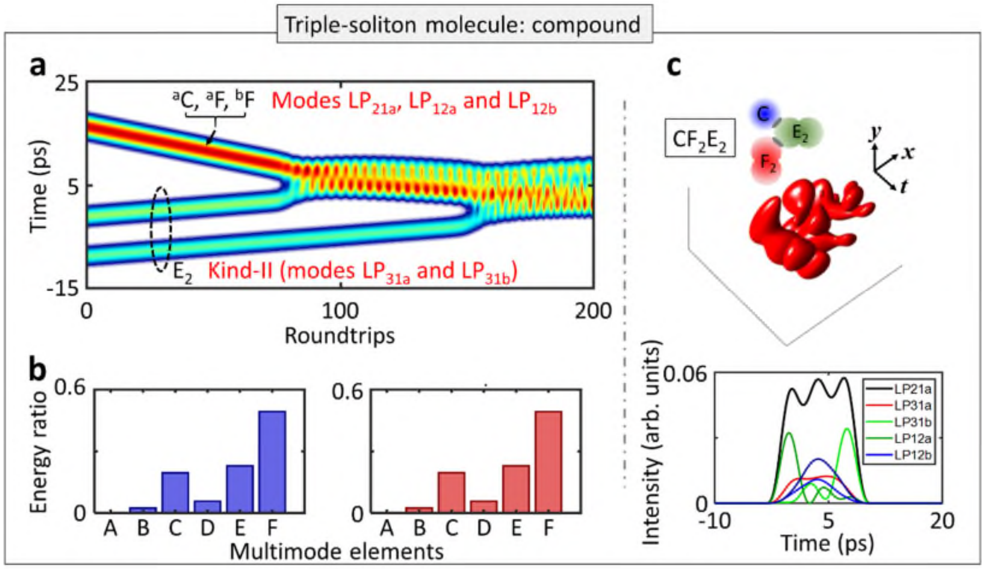

Supplementary Figure 25 | Temporal dynamics of 3D triple-soliton molecules. a. Temporal evolution of the 3D triple-soliton molecule. **b**. Energy distributions of the multimode elements before (left) and after (right) the formation of the 3D triple-soliton molecule. **c**. Visualization of the 3D triple-soliton molecule. (Top) Isosurface plot of the 3D triple-soliton molecule. (Bottom) Mode-resolved intensity profiles on the time-intensity plane.

3D triple-soliton molecule

The generation of 3D triple-soliton molecules in the ST mode-locking is much more complicated due to the modal/spatial complexity[11]. To show this, we present typical assembling dynamics in **Supplementary Figure 25**. As illustrated, in the initial state, a single 3D soliton that involves LP_{21a}, LP_{12a} and LP_{12b} modes approaches a kind-II dual-soliton molecule (i.e., a homonuclear diatomic molecule involving LP_{31a} and LP_{31b}). As depicted in **Supplementary Figures 25a,b**, the production of the 3D triple-soliton molecule, as a hybrid case of kind-I and -II dual-soliton molecules, undergoes a similar binding process as that of the 3D dual-soliton molecule (**Supplementary Figure 24**). Such assembling dynamics can emulate a combination reaction of generating a compound, as the invariant modal energy distribution can serve as the analogue of the law of conservation of mass. According to the mode-resolved intensity

profiles shown in **Supplementary Figure 25c**, the 3D triple-soliton molecule can be treated as an analogous molecular formula CF_2E_2.

7.3. Underlying dynamic rules of the soliton molecular dynamics

To clarify the underlying rules of the soliton molecular dynamics, we further investigate the assembling dynamics of a kind-I dual-soliton molecule (i.e., a heteronuclear diatomic molecule CE), as shown in **Supplementary Figure 26**.

Three phases of the assembling dynamics are included, as illustrated in **Supplementary Figure 26b**. In phase 1 (P1, i.e., dissociated atoms), the pulses in LP_{21a} and LP_{31a} modes propagate at different group velocities (see in **Supplementary Figure 26a**), and there is no spatiotemporal overlapping (see P1 in **Supplementary Figure 26b**), leading to weak nonlinear-interaction-induced binding force. Thus, the pulses in LP_{21a} and LP_{31a} modes behave like dissociated atoms, i.e., elements ^{a}C and ^{a}E. The existence of group velocity mismatch, primarily caused by the modal and chromatic dispersion (with ~0.04 THz frequency difference), can impose weak attraction on the atoms and thus initiate the self-organization of the 'CE' molecule.

In phase 2 (P2, i.e., binding process), the desynchronized pulses overlap in the time and space domains (see the arrow in the subplot P2 of **Supplementary Figure 26b**) and thus nonlinearly interact to produce molecular-like binding force between the initially uncorrelated atoms, i.e., dissociated elements ^{a}C and ^{a}E. As shown in **Supplementary Figure 26c**, the binding process (like reaction) starts from roundtrip ~700 and lasts for ~360 roundtrips, during which a maximum strength of $f = 4.3 \times 10^{-4}$ is generated.

In phase 3 (P3, i.e., molecule formation), the equilibrium of the reaction attains is established, i.e., $d\langle\omega_1\rangle/dN = 0$, leading to the successful assembling of the heteronuclear diatomic molecule 'CE', whose intensity profiles on the time-intensity plane before and after the assembling are shown in **Supplementary Figure 26d**.

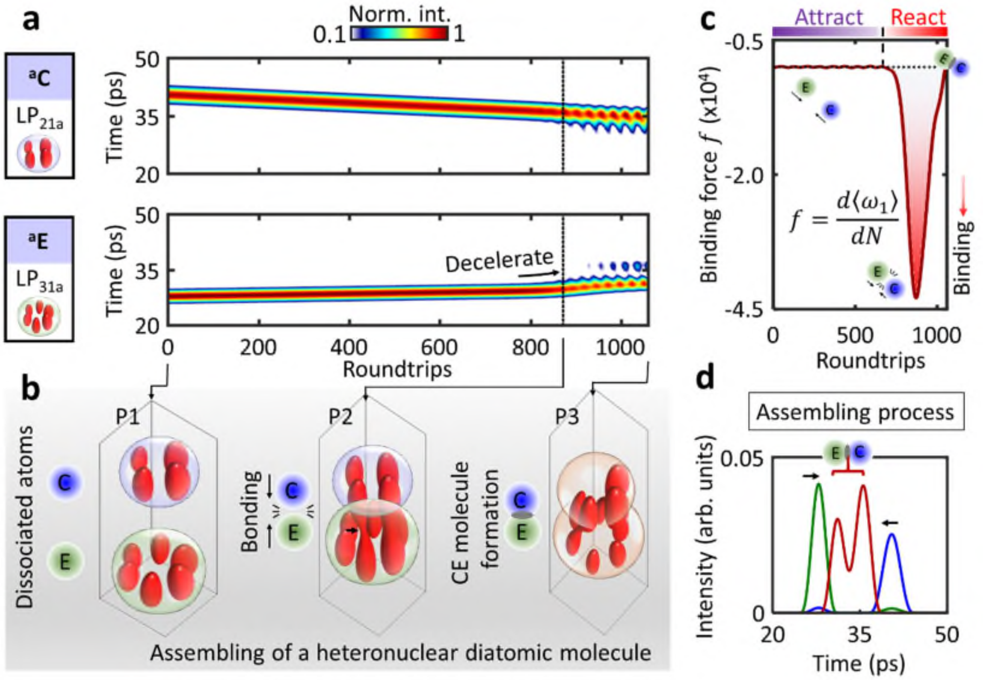

Supplementary Figure 26 | 3D soliton molecular dynamics analogous to the assembling of heteronuclear diatomic molecule. a. Temporal evolutions of elements ^{a}C (LP_{21a}, top) and ^{a}E (LP_{31a}, bottom). **b**. Three phases of assembling dynamics of ‘CE’ molecule. The spatiotemporal interaction between elements C and E is intuitively illustrated by isosurface plots of the multimode intensity profile. **c**. Binding force. **d**. Intensity profiles on the time-intensity plane before and after the assembling (corresponding to roundtrips 1 and 1060).

Supplementary Figure 27 further shows the 3D soliton molecular dynamics with greater complexity. In our simulation, because of the modal/spatial complexity, we observe different 3D soliton molecules in a long-term assembling process (~30,000 roundtrips), as illustrated in **Supplementary Figure 27a.** During the assembling process, different soliton molecules are discovered (**Supplementary Figure 27b**), which can be analogous to multiple intermediates produced in a complex combination reaction. In the initial state, a pair of pulses in LP_{31a} and LP_{31b} modes co-propagate as the kind-II soliton molecule, this dual-soliton molecule can be treated as an analogous molecular formula, i.e., E_2. Then, a single 3D soliton that involves LP_{21a}, LP_{12a} and LP_{12b} modes (indicated by the blue arrow in **Supplementary Figure 27a**) approaches

the homonuclear diatomic molecule E_2, resulting in the production of a 3D triple-soliton molecule CF_2E_2. Finally, by further reacting with a single soliton in LP_{21b} mode, as indicated by the red arrow in **Supplementary Figure 27a**, a stable molecule $C_2F_2E_2$ is produced.

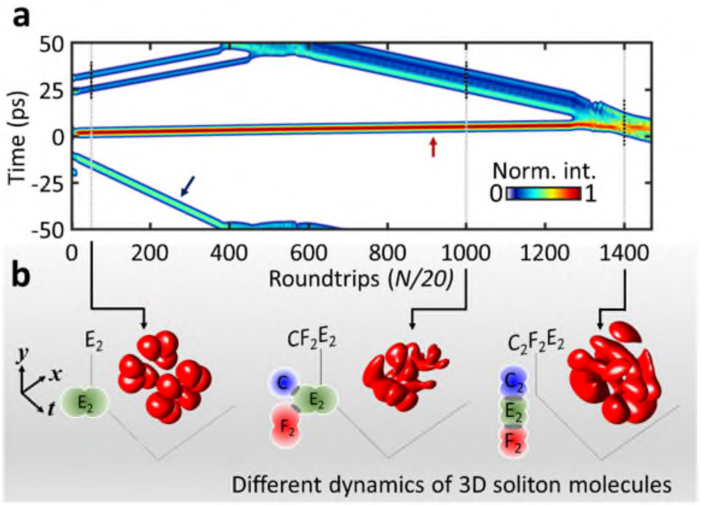

Supplementary Figure 27 | 3D soliton molecular assembling with greater complexity. a. Temporal evolution calculated from the scalar superposition of the first 10 transverse modes. **b**. Three different 3D soliton molecules, i.e., E_2, CF_2E_2, and $C_2F_2E_2$ shown as isosurface plots.

Here, we address that the assemblings of dual-soliton or triple-soliton molecules are usually susceptible to the initial modal condition (i.e., 3D initial condition). This reminds us of the versatile spectral-temporal evolution of single-passing the parabolic multimode optical fiber by controlling the input spatial profile[12]. Hence, to access different ST soliton molecules, we usually use different initial conditions.

Supplementary References

1. F. Poletti, P. Horak, Description of ultrashort pulse propagation in multimode optical fibers. *J. Opt. Soc. Am. B* **25**, 1645-1654 (2008).
2. L. G. Wright, P. Sidorenko, H. Pourbeyram, Z. M. Ziegler, A. Isichenko, B. A. Malomed, C. R. Menyuk, D. N. Christodoulides, F. W. Wise, Mechanisms of spatiotemporal mode-locking. *Nat. Phys.* **16**, 565-570 (2020).
3. R. Kubo, M. Toda, N. Hashitsume, Statistical physics II: nonequilibrium statistical mechanics. *Springer Science & Business Media* (2012).
4. X. Liu, X. Yao, Y. Cui, Real-time observation of the buildup of soliton molecules. *Phys. Rev. Lett.* **121**, 023905. (2018).
5. K. K. Tsia, K. Goda, D. Capewell, B. Jalali, Performance of serial time-encoded amplified microscope. *Opt. Express* **18**, 10016-10028 (2010).
6. W. H. Renninger, F. W. Wise, Optical solitons in graded-index multimode fibres. *Nat. Commun.* **4**, 1-6. (2013).
7. L. G. Wright, D. N. Christodoulides, F. W. Wise, Controllable spatiotemporal nonlinear effects in multimode fibres. *Nat. Photon*. **9**, 306-310 (2015).
8. L. G. Wright, W. H. Renninger, D. N. Christodoulides, F. W. Wise, Spatiotemporal dynamics of multimode optical solitons, *Opt. Express* **23**, 3492-3506 (2015).
9. A. Hause, H. Hartwig, M. Böhm, F. Mitschke, Binding mechanism of temporal soliton molecules. *Phys. Rev. A* **78**, 063817 (2008).
10. Y. Guo, X. Wen, W. Lin, W. Wang, X. Wei, Z. Yang, Real-time multispeckle spectral-temporal measurement unveils the complexity of spatiotemporal solitons. *Nat. Commun.* **12,** 1-9 (2021).
11. M. A. Eftekhar, Z. Sanjabi-Eznaveh, H. E. Lopez-Aviles, S. Benis, J. E. Antonio-Lopez, M. Kolesik, F. Wise, R. Amezcua-Correa, D. N. Christodoulides, Accelerated nonlinear interactions in graded-index multimode fibers. *Nat. Commun.* **10**, 1-10 (2019).
12. M. A. Eftekhar, L. G. Wright, M. S. Mills, M. Kolesik, R. A. Correa, F. W. Wise, D. N. Christodoulides, Versatile supercontinuum generation in parabolic multimode optical fibers. *Opt. Express* **25**, 9078-9087 (2017).
